# Heterogeneity of Fish Taxonomic and Functional Diversity Evaluated by eDNA and Gillnet along a Mangrove–Seagrass–Coral Reef Continuum

**DOI:** 10.3390/ani13111777

**Published:** 2023-05-26

**Authors:** Shuting Qiu, Jillian Lean Sim Ooi, Weilin Chen, Sze-Wan Poong, Han Zhang, Weiyi He, Shangke Su, Hao Luo, Wenjia Hu, Yang Amri Affendi, Jianguo Du, Kar-Hoe Loh

**Affiliations:** 1 Institute of Ocean and Earth Sciences, Universiti Malaya, Kuala Lumpur 50603, Malaysia; 2 Key Laboratory of Marine Ecological Conservation and Restoration, Third Institute of Oceanography, Ministry of Natural Resources, Xiamen 361005, China; 3 Institute for Advanced Studies, Universiti Malaya, Kuala Lumpur 50603, Malaysia; 4 Department of Geography, Faculty of Arts and Social Sciences, Universiti Malaya, Kuala Lumpur 50603, Malaysia; 5 Faculty of Marine Biology, Xiamen Ocean Vocational College, Xiamen 361100, China

**Keywords:** environmental DNA, fish community, functional diversity, mangrove, seagrass, coral reef

## Abstract

**Simple Summary:**

The selection of fish community survey methods based on their efficiency and reliability is critical for ecosystem conservation, protection and management. In this study, we used environmental DNA (eDNA) metabarcoding and traditional (gillnet) fishing to assess the diversity of fishes along a mangrove–seagrass–coral reef continuum of marine habitats in Hainan, China. Higher fish taxonomic diversity was identified using eDNA; however, gillnet fishing was better at identifying fish communities from different habitats along the sampled continuum. Results from both survey methods indicate that some fish species use multiple habitats along mangrove–seagrass–coral reef continuums. Therefore, the concurrent use of eDNA and gillnet survey methods provides a more comprehensive approach to understanding the heterogeneity of fish taxonomic and functional diversity along mangrove–seagrass–coral reef continuums.

**Abstract:**

The effective and reliable monitoring of fish communities is important for the management and protection of marine ecosystems. Environmental DNA (eDNA) metabarcoding is a relatively new method that has been widely used in recent years, while traditional sampling via fish catching (i.e., gillnets) is one of the most common and reliable fish monitoring methods used to date. We compared the taxonomic and functional diversity of fish detected within a mangrove–seagrass–coral reef continuum using both survey methods. One liter seawater and gillnet samples were collected in August 2021 from mangrove forests, seagrass meadows and coral reef habitats (*n* = 3 each) in Hainan, China. Surveys using eDNA and gillnets identified 139 genera belonging to 66 families and 58 genera belonging to 42 families, respectively. Regardless of the survey method, fish detected in mangrove, seagrass and coral reef habitats were heterogeneous in their communities; however, the shared species between habitats suggest some degree of connectivity. There were no significant differences between habitats in terms of taxonomic and functional diversity, but a higher taxonomic diversity was detected using eDNA. Both methods were able to distinguish fish assemblages between different habitats; however, gillnet surveys performed better than eDNA surveys for distinguishing mangrove from seagrass assemblages. Therefore, the concurrent use of eDNA and gillnet survey methods provides a more comprehensive approach to understanding the heterogeneity of fish taxonomic and functional diversity along mangrove–seagrass–coral reef continuums.

## 1. Introduction

Mangroves, seagrass meadows and coral reefs usually coexist in the marine tropics. These marine ecosystems interact chemically, biologically and physically, contributing to nutrient cycling via biogeochemical cycles, and facilitate nutrient exchange between trophic levels [1]. Thirty-five percent of the world’s mangroves have disappeared, and they are still degrading at an average rate of 0.99% per year despite recent improvements in the enhanced restoration and reduction of deforested areas [2,3]. The degradation of seagrass habitats, estimated to be at a rate of 1.5% per year during 1990–2006 [4], is of similar concern. The world’s cumulative degraded or lost seagrass habitat area ranges from 177,000 to 600,000 km^2^ or more [5]. As for coral reef habitats, 27% of these areas have suffered decimation [6], whilst for the remaining coral reefs, 72% are under local (e.g., pollution) and global (e.g., climate change) pressures to survive [7]. The health of coastal habitats is closely linked to the creatures that depend on them [1], such as fish, which have commercial, recreational and cultural importance [8]. Therefore, it is necessary that more research is undertaken to improve our understanding of mangrove, seagrass meadow and coral reef ecosystems and the fish that rely on them.

The key to assessing the diversity of costal habitats is effective and authentic biomonitoring, which is also essential for policy formulation and the implementation of conservation and restoration projects [9]. Traditional biomonitoring (e.g., gillnets) is the most routinely used mainstream method for fish surveys, and has been found to be reliable [10,11]. However, there are limitations to the gillnet method, such as higher sampling costs in terms of funds and time, a higher dependence on taxonomic expertise and a greater probability of neglecting cryptic or small species [12,13,14]. Hence, it is necessary to carry out sampling in tandem with other methods such as the use of eDNA, which offers the advantages of high cost-efficiency and non-invasiveness and enables the sampling of a relatively wider distributional range [12,15]. Studies have shown that combining eDNA with traditional survey methods can reflect the status of local fish communities more accurately [16,17], and the small-scale heterogeneity of fish diversity can also be evaluated using eDNA [18].

Hitherto, most ecological protection and restoration policies have been formulated based on a single dimension of biodiversity, while neglecting other indicators of diversity with more dimensions (e.g., functional diversity) [19,20]. Animals perform different functions in the ecosystem that are closely related to the ecosystem’s structure and function [21]. The adaptations of these animals to different ecosystems are called functional traits, including—but not limited—to morphology, behavior and physiology [22]. Functional diversity is an index that reflects the value and range of functional traits in a community or ecosystem; it is considered a reliable metric to explore the relationships between fish community structures and ecosystem functioning in their habitats [23]. The combination of functional diversity and traditional taxonomic diversity indicators has been found to provide a more comprehensive assessment of local fish community structures—thereby strengthening the effectiveness of policies for protection and restoration [24,25]. However, research on eDNA and functional diversity of fish in mangrove–seagrass meadow–coral reef continuums is mostly concentrated in the Caribbean region, while there is a lack of studies on tropical fish diversity in the South China Sea [26,27].

The Qinglangang Nature Mangrove Reserve is situated in Wenchang, on the east coast of Hainan Province, China. This reserve accommodates the largest number of man-grove species [28] and the largest area of seagrass in China [29], with a certain amount of coral reef [30], making it a rare continuum of mangrove, seagrass meadow and coral reef habitats within the country [31]. In recent years, these three habitats have suffered degradation to varying degrees [30,32,33] due to human activities such as marine pollution and land reclamation and natural causes such as typhoons. To ensure the effective protection and restoration of these varied ecosystems, it is essential to provide accurate biological monitoring data from different habitats. The present study aimed to (i) determine the ability of data collected using eDNA metabarcoding and gillnet methods to differentiate fish assemblages among adjacent mangrove, seagrass meadow and coral reef habitats and (ii) explore the heterogeneity of fish in different habitats. The results provide theoretical support and constructive suggestions for the selection of field survey methods and data for effective ecosystem management and conservation.

## 2. Materials and Methods

### 2.1. Study Area

**Figure 1:**
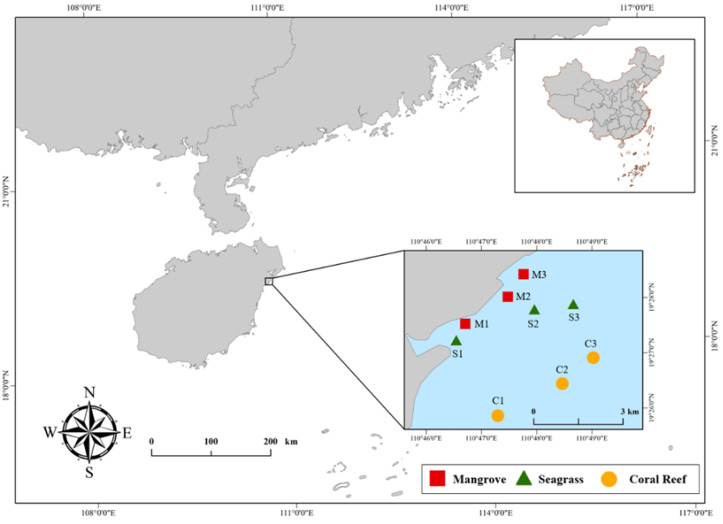
Three sections of the mangrove–seagrass–reef continuum in Wenchang, Hainan Province, China. Mangrove sampling sites were on average close to 400 m offshore, with coral reefs on the marginal continental shelf and seagrass meadow in between.

Wenchang has a tropical marine monsoon climate with distinct dry and rainy seasons and climate characteristics such as ample sunlight, high temperatures and abundant rainfall [34]. Seawater and gillnet samples were collected in August 2021 from three sampling sites for each of the habitats along the targeted mangrove–seagrass–coral reef continuum (Figure 1). During each sampling, the seawater sample was collected first, followed by the gillnet sample. In the mangrove area, the sediments were mainly composed of sand and silt and the dominant mangrove species were *Aegiceras corniculatum*, *Bruguiera gymnorrhiza* and *Kandelia candel* [35]. The sediments in the seagrass meadow mainly consisted of coral chips, shell chips and gravel, with the dominant seagrass species being *Halophila ovalis* and *Enhalus acoroides*. In recent years, due to the discharge of wastewater from surrounding aquaculture and other reasons, the distribution of seagrass in Wenchang has shown an obvious trend of degradation that has caused the seagrass meadows to become patchy or scattered. Seagrass areas have reportedly reduced from 31.8 km^2^ in 2012 to 18.8 km^2^ in 2022 [29,33]. The dominant coral genera from the reefs were *Porites*, *Favites* and *Platygyra*. The average coverage of live scleractinian (hard) coral, soft coral and macroalgae was 4.4%, 0.1% and 22.0%, respectively, and the hard coral recruitment was 0.2 ind/m^2^ [30].

### 2.2. Environmental DNA Sampling and Library Construction of 12S-rDNA Barcodes

A 1 L sterile bag was used to collect surface water at each site (three times) and was immediately stored on ice before deploying the gillnet (see Section 2.3). After sampling, the entire 1 L water sample collected was filtered through a 0.45 μm Pall Corporation membrane using a vacuum pump and a polyphenylsulfone filter cup. The surface of the workbench and each piece of utilized equipment was thoroughly cleaned before and after each use with distilled water to avoid cross-contamination of samples [16]. After the filtering process was completed, each filter membrane was placed into a 1.8 mL cryovial tube and stored on dry ice until transportation back to the laboratory at the Third Institute of Oceanography. Captured eDNA was extracted from the filter membranes using the DNeasy Blood and Tissue Kit (Qiagen, Germany). Recovered total DNA for all samples was then sent to Sango Biotech (China) for PCR and sequencing. The taxonomic information and distribution records for each fish species in this study were obtained from FishBase.org [36]. For each fish species, five (or otherwise all available) 12S-rDNA gene sequences were downloaded from the National Center for Biotechnology Information (NCBI) [https://www.ncbi.nlm.nih.gov/, accessed on 15 June 2022] NT (Nucleotide) database as a reference for the mapping sequencing reads. The 12S-rDNA MiFish-U primer pair [37] (MiFish-UF 5′: GTC GGT AAA ACT CGT GCC AGC; MiFish-UR 3′: CAT AGT GGG GTA TCT AAT CCC AGT TTG) was used because the 12S-rDNA gene has been demonstrated to be useful for detecting fish diversity and the MiFish-U primer pair has been shown to have high discriminatory power [38,39]. The 30 μL PCR reaction included 15 µL of Taq 2x Hieff^®^ Robust PCR Master Mix (Yeasen, China), 1 μL of 10 μM forward and reverse primers, 1 μL of DNA template, and 12 μL of sterile distilled H_2_O. Thermal cycling included an initial denaturing step of 96 °C for 2 min, 35 cycles of 95 °C for 30 s of denaturation, 60 °C for 30 s of annealing, 72 °C for 40 s of extension, and a final extension step of 72 °C for 5 min. A 2% agarose gel was used to check for target bands. To obtain high-quality sequencing data, only samples with a DNA concentration above 10 ng/μL, as assessed by a Qubit 4.0 fluorometer, were sequenced. The sequencing depth was standardized to 60,000 reads for each sample. After sequencing, the two short Illumina reads were assembled using PEAR software (version 0.9.8) [40], based on the overlap. Subsequently, the fastq files were processed to generate individual fasta and qual files. The effective tags were clustered into operational taxonomic units (OTUs) of ≥97% similarity using Usearch software (version 11.0.667) [41]. Singleton OTUs (only one read) and chimeric sequences were excluded, and the remaining sequences were grouped into each sample based on the determined OTUs. The tag sequence with the highest abundance was selected to represent each cluster.

### 2.3. Gillnet Sample Collection and Species Identification

Traditional fish sampling was conducted concurrently with eDNA sampling (see Section 2.2) in August 2021 using gillnets. Gillnets (1 m high and 150 m wide; 0.5 cm mesh size) were used in all three habitats and deployed for 1 h. Each sampling was repeated five times. Fish were driven toward the gillnet by manually beating the water surface. All fish captured during gillnet sampling were counted and identified to the minimum taxonomic level possible according to local species identification guides [42,43].

### 2.4. Data Analysis

Species-level identification was not possible for some of the fish captured by gillnet sampling due to the limited resolution of the NCBI nucleotide database. As such, eDNA analyses were conducted at the genus level to include as much data as possible and permit comparisons across sampling methods (i.e., eDNA versus gillnet). Gillnet count data and eDNA OTU abundance data were log-transformed (x + 1). Differences in genera composition between the three habitats and two sampling methods were tested by permutational analysis of variance (PERMANOVA). The genera accumulation curve was extrapolated by the R package iNEXT to reflect the representativeness of the sampling stations in this survey [44]. Principal coordinate analyses (PCoAs) were also used to explore the heterogeneity of fish in different habitats and to determine the ability of both methods to differentiate fish assemblages among adjacent mangrove, seagrass and coral reef habitats (i.e., across continuums). The SIMPER (Similarity Percentage) analysis was used to screen the fish genera that contributed the most to habitat heterogeneity and to differences between methods [45]. For each sampling site, the taxonomic diversity was measured by the fish richness and the functional diversity was represented by the richness (FRic). The FRic refers to the size of the functional space occupied by an organism in a community, which reflects an index of the degree of ecological space utilization [24]; a larger index indicates a higher degree of ecological space utilization. To analyze the taxonomic and functional diversity, the mixed linear model was employed to compare the effects of fixed and random factors, followed by Tukey’s HSD post-hoc test when the differences were significant (*p* < 0.05). PERMANOVA, PCoAs, SIMPER analysis and taxonomic diversity were all calculated using the R package Vegan [46], while functional diversity was calculated using the R package mFD [47] and visualization was performed using the R package ggplot2 [48].

A combination of six traits (Trophic Level, Maximum Total Length, Water Column Position, Mobility, Habitat Type and School Size) that are able to well describe the functions performed by fish species was chosen in this study. The description of functional traits and categories and functional trait matrix for the six functional traits is attached to the Supplementary Materials (Tables S1 and S2). The selected traits are mainly related to fish diet, movement and habitat use. Information was obtained from the online databases FishBase [36] and the Fish Database of Taiwan [49].

## 3. Results

### 3.1. Species Accumulation Curves

A total of 3,809,039 raw paired-end reads were obtained from 24 samples subjected to eDNA metabarcoding using MiFish-U primers, with 3,452,506 (90.1%) remaining after filtering, chimera removal and processing. A seawater sample from one of the seagrass meadow sites (S3) and two seawater samples from the coral reef sites (C1 and C2) failed during PCR amplification and were excluded from sequencing and subsequent analyses. Thirty-seven (23.2%) fish genera were detected by both eDNA and gillnets, while an additional 102 (63.8%) and 21 (13.1%) genera were resolved by eDNA and gillnets, respectively. At the family level, there were 32 (43.2%) common fish families detected using both methods, whereas an additional 32 (43.2%) and 10 (13.6%) families were identified with eDNA and gillnets, respectively. The number of fish genera detected by eDNA was significantly higher than that detected by gillnets (Mann–Whitney–Wilcoxon Test, W = 1812, *p*-value < 0.01).

**Figure 2:**
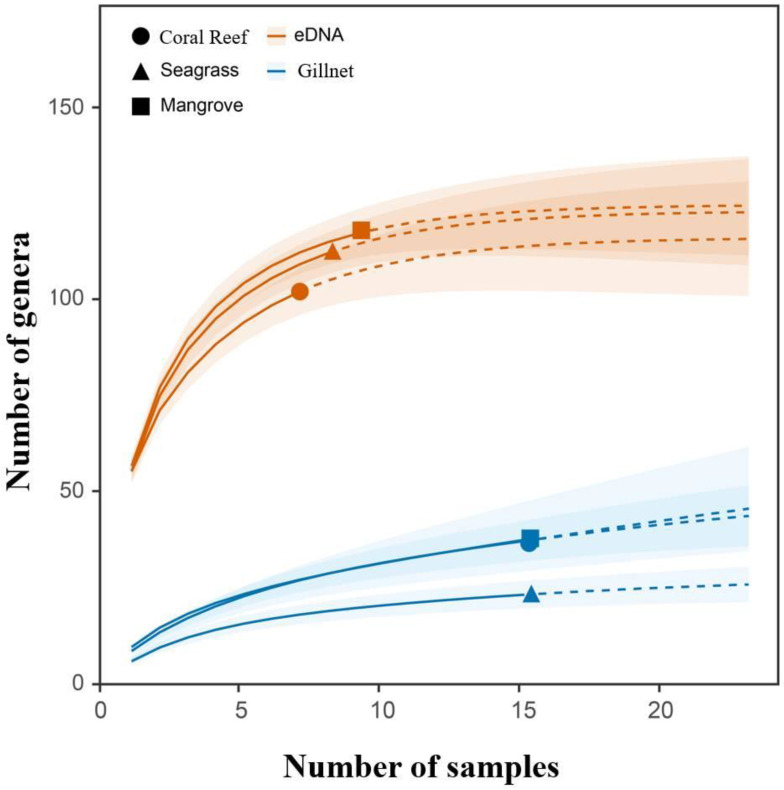
Species accumulation curves for the number of fish genera detected at Wenchang, Hainan in South China using eDNA metabarcoding and gillnets along a mangrove–seagrass–coral reef continuum. The solid line represents the measured value of the survey while the dotted line represents the predicted value of the INEXT package, and the shaded part denotes the 95% confidence interval for the prediction.

Surveys using eDNA metabarcoding recorded 93.6%, 91.1% and 88.9% of the genera asymptote in the mangrove (117 genera), seagrass (112 genera) and coral reef (102 genera) habitats, whereas gillnet surveys recorded 90.2%, 85.2% and 78.7% of genera asymptote in the mangrove (37 genera), seagrass (23 genera) and coral reef (27 genera) habitats (Figure 2). Overall, eDNA surveys detected fewer coral reef fish genera than seagrass fish genera, which is contrary to the observed results from the gillnet surveys, whereas both the eDNA and gillnet surveys detected more mangrove fish genera than seagrass and coral reef fish genera.

### 3.2. Fish Taxonomic Information

**Figure 3:**
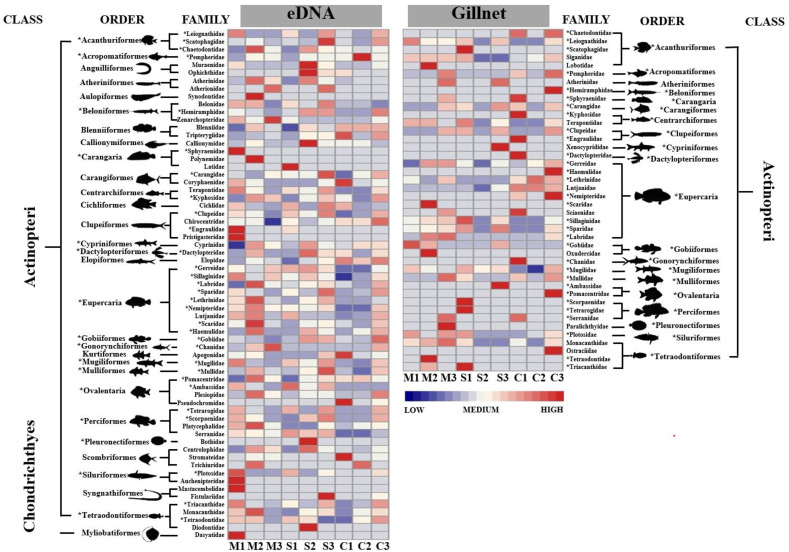
Heatmap of fish community structure detected from samples collected using eDNA and gillnets. Asterisks (*) indicate the common genera and families surveyed by the two methods. M1–3, S1–3 and C1–3 refer to the three mangrove, seagrass meadow and coral reef sampling sites across the habitat continuum, respectively (see Figure 1). Heatmap colors represent the relative abundance of the detected fish families: blue indicates low abundance, white indicates moderate abundance and red indicates high abundance.

The analysis of the eDNA metabarcoding results indicated the presence of two fish classes divided into 30 orders, 66 families and 139 genera, with Eupercaria as the dominant order (Figure 3). In contrast, the analysis of the gillnet results indicated the presence of one fish class divided into 20 orders, 42 families and 58 genera, with Eupercaria as the dominant order. Eighty-seven (59.7%) fish genera and forty-six (70.8%) families were detected across the mangrove–seagrass–coral reef continuum using eDNA, whereas 12 (20.7%) common fish genera and 13 (31%) fish families were detected across the same continuum using gillnets.

**Figure 4:**
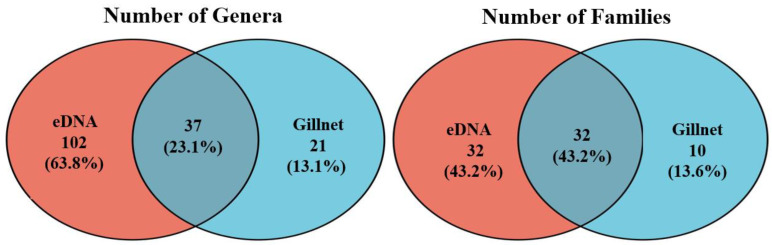
Venn diagram depicting the number of fish genera and families identified using eDNA, gillnets and both methods across the sampled mangrove–seagrass–coral reef continuum.

A comparison of the two methods found that the genera and families jointly identified by eDNA and gillnets were 37 and 32, which accounted for 23.1% and 43.2% of the total, respectively (Figure 4). It is worth noting that despite being detected by both sampling methods, the relative abundance of fish detected from the Haemulidae, Scorpaenidae, Tetrarogidae, Tetraodontidae and Triacanthidae families was higher for gillnets than eDNA.

### 3.3. Fish Communities

**Figure 5:**
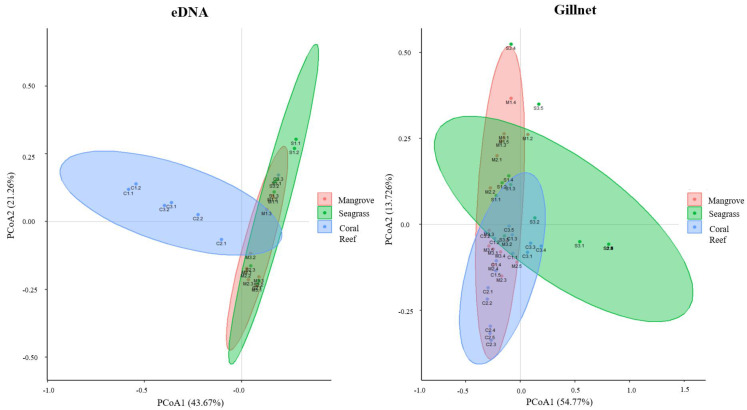
Results of Principal Coordinates Analysis showing the relationship of fish (genera) assemblages identified in each sample from Wenchang, Hainan in South China using habitat (mangrove vs. seagrass vs. reef) and method (eDNA vs. gillnet) as factors.

The composition of fish genera was significantly different between eDNA and gillnet samples (*p* < 0.001) and among mangrove, seagrass and coral reef habitats (*p* < 0.001; Table 1). A clear separation in ordination could be seen for fish genera detected across the sampled mangrove–seagrass–coral reef continuum for the gillnet samples despite some overlaps, whereas the observed overlap between the mangrove and seagrass was larger for the eDNA samples (Figure 5). This demonstrates the higher discriminatory power of gillnets versus eDNA for discriminating fish genera in different habitats—especially between mangroves and seagrass meadows. Moreover, these observations indicate that the fish composition across the sampled mangrove–seagrass–coral reef continuum was composed of species endemic to that habitat, as well as common species distributed in two or three of the habitats, which suggests that some fish genera have connectivity across the sampled continuum.

SIMPER analysis revealed that *Plotosus* (70.3%), *Triacanthus* (67.8%) and *Leiognathus* (65.0%) were the genera that contributed the greatest dissimilarity between the detected fish assemblages using eDNA and gillnets; however, all three of these fish genera were detected using both sampling approaches. For the eDNA method, the SIMPER analysis revealed that (1) the *Capoeta* (71.7%), *Pelates* (68.5%) and *Mugil* (64.7%) genera accounted for the greatest dissimilarity between the mangrove and seagrass habitats; (2) the *Plotosus* (72.0%), *Capoeta* (69.5%) and *Triacanthus* (66.6%) genera accounted for the greatest dissimilarity between the seagrass and coral reef habitats; and (3) *Capoeta* (72.2%), *Plotosus* (69.5%) and *Terapon* (66.6%) accounted for the greatest dissimilarity between the mangrove and coral reef habitats. For the gillnet method, the SIMPER analysis revealed that (1) *Terapon* (70.1%), *Eubleekeria* (64.2%) and *Lutjanus* (58.9%) accounted for the greatest dissimilarity between the mangrove and seagrass habitats; (2) *Terapon* (74.4%), *Gerres* (67.6%) and *Eubleekeria* (58.8%) accounted for the greatest dissimilarity between the seagrass and coral reef habitats; and (3) *Plotosus* (73.6%), *Terapon* (69.7%) and *Pelates* (65.1%) accounted for the greatest dissimilarity between the mangrove and coral reef habitats (Table 2).

### 3.4. Taxonomic Diversity and Functional Diversity

**Figure 6:**
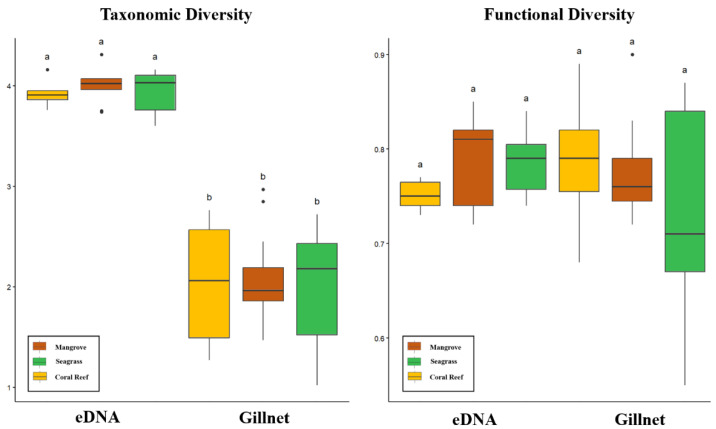
Detected taxonomic diversity and functional diversity using eDNA and gillnet survey methods in three habitats across a mangrove–seagrass–coral reef continuum in Wenchang, Hainan, China. (Letters a and b represent significance levels. If there is a significant difference between groups, it will be denoted by a or b. Otherwise, the same letter will be used to represent them uniformly.)

Between survey methods, the detected taxonomic diversity of the fish was significantly higher for eDNA than gillnets for all three habitats within the sampled mangrove–seagrass–coral reef continuum (Tukey HSD post-hoc, *p* < 0.05); however, within the survey methods, the detected taxonomic diversity was consistent (*p* > 0.05) across the sampled continuum habitats (Figure 6). Functional diversity exhibited no significant differences (*p* > 0.05) between the eDNA and gillnet survey methods or the sampled continuum habitats, regardless of the survey method. This indicates that eDNA is more useful in assessing taxonomic diversity than gillnets; however, eDNA and gillnets exhibited a comparable ability to distinguish taxonomic and functional diversity between the different habitats within the sampled continuum.

## 4. Discussion

Global fish surveys are increasingly relying on the use of multiple survey approaches to monitor and distinguish fish assemblages for a better understanding of actual marine and fishery health conditions. In this study, eDNA and gillnet (i.e., traditional fish capture) survey methods were used to explore their ability to differentiate fish assemblages along a mangrove–seagrass–coral reef continuum and to analyze the habitats’ heterogeneity using taxonomic diversity and functional diversity metrics.

We found that the results of combining eDNA (i.e., contemporary) and gillnet (i.e., traditional) survey methods show high complementarity, rather than one method being superior to the other. This is attributed to each survey method having inherent selectivity and limitations [50]; for example, the gillnet method presents a high possibility of overestimating the abundance of fish species that are large bodied and that swim faster [51]. Additionally, the likelihood of underestimating smaller-bodied fish increases with the gillnet mesh size [52]. In contrast, the eDNA method can have sampling bias due to incomplete reference databases that prevent distribution information from being included for some species [53]. For example, in this study, *Johnius dussumieri*, *Leiognathus berbis*, *Nuchequula mannusella*, *Ostracion cubicum* and *Pennahia aneus* were only identified with gillnets due to a lack of 12S-rDNA sequence data for these species in the NCBI GenBank database. Another sampling bias inherent to the eDNA method is the different amplification preference of the primers, which means that sequences from certain fish families were preferentially amplified compared to sequences from other fish families [54]. A possible cause for the different abundance of the same fish genera between the eDNA and gillnets in this study is behavior, given that eDNA has been demonstrated to exhibit a higher recognition rate for some species (e.g., predatory or pelagic fish) because they leave more copy numbers in the water column [8]. Therefore, it is reasonable for fish from the families Haemulidae, Scorpaenidae, Tetrarogidae, Tetraodontidae and Triacanthidae, which generally occupy the bottom of the ocean and are slower moving, to present a lower relative abundance when detected with eDNA compared to gillnets [55].

Our results showed that the number of genera and taxonomic diversity detected by eDNA were significantly higher than detection by gillnets, which could be attributed to the tendency of DNA fragments to persist longer in sediment than in water (i.e., the legacy versus the temporally-restricted presence of target species within sampled habitats) [56]. This is despite precautionary measures taken during sampling by collecting water samples prior to gillnet fishing to minimize the impact of resuspended sediment DNA on species detection. The data from the current study indicate that the highest number of fish genera in both survey methods was detected in the sampled mangrove habitats. This is inconsistent with results in mangrove–seagrass–coral reef continuums in other global regions—specifically Puerto Rico and the Philippines—which have shown that the number of species detected in coral reef habitats within the studied continuums was the highest, while the mangrove and seagrass habitats within the studied continuums acted as nurseries [57,58]. Nonetheless, the results of this study corroborate with past studies from the same geographic area (i.e., China) [31], supporting the notion of our data reflecting the real local fish structure across the sampled mangrove–seagrass–coral reef continuum. The poor condition of the sampled seagrass meadows and coral reefs may be the main reason for the observed lower species abundance compared to mangroves. Due to pollution from nearby aquaculture plants, the seagrass meadows in Wenchang have decreased significantly in species diversity and coverage area, gradually becoming patchy and scattered [27]. Similarly, the coral reefs in Wenchang are facing the pressure of land reclamation from the sea, and the number of coral reef species, coverage and replenishment and the surrounding water quality have significantly decreased over recent years [30]. This signals the urgent need to strengthen the protection and restoration of coastal ecosystems in Hainan and suggests that the designation of protected marine areas should be carried out with as much consideration of fish connectivity as possible by viewing the different habitats along the mangrove–seagrass–coral reef continuum as interconnected rather than isolated ecosystems.

Our study provides strong support for using eDNA to assess fish community structures in different habitats, which has also been demonstrated in other studies [15,59,60]. The results of the PCoA showed that regardless of the sampling method, there was a certain amount of overlap between the mangrove, seagrass meadow and coral reef habitats—implying a connection between these three continuum habitats, which is consistent with a previous habitat continuum eDNA study [31]. However, given that the biological information of the fish such as the body length and weight cannot be elucidated from the eDNA method, further analyses of habitat utilization by detected fish are not possible. Environmental DNA as a survey method may be very efficient and fast for collecting species and community data in marine investigations targeting multiple habitat types (e.g., continuum or non-continuum); however, traditional gillnet surveys can provide more detailed information about fish communities such as sex ratio and age structure, which are important parameters for community analysis [58]. Nonetheless, with the development and improvement of eDNA methods, these metrics may be able to be assessed in the future [61]. In this study, we observed that eDNA surveys could distinguish fish assemblages within sampled mangrove and seagrass habitats less effectively than gillnet surveys (Figure 5). This is probably due to the <500 m distance between the sampled mangrove and seagrass habitats, in that eDNA can be transported over such distances via tides and currents (i.e., are more likely to impact habitat discrimination for eDNA samples than gillnet samples) [15,62].

To maximize the efficiency and accuracy of species detection using eDNA in different habitats, improvements and additional efforts in field sampling design and laboratory techniques may be required [63]. For example, studies have demonstrated that the degradation of eDNA can be influenced by filter pore size, water temperature, the target gene and the water source—not as individual parameters, but rather in combination [64,65]. Additionally, the shedding rate and excretion rate of eDNA signals are determined by many factors [66,67], and this may be the one of the reasons affecting the ability of eDNA to identify fish assemblages in different habitats. Increased sampling frequency has been shown to help reduce sampling randomness and provide a more comprehensive understanding of local fish communities [68]. Therefore, it is important to find a balance between sampling cost and efficiency.

The south sea of China has diverse ecosystems and rich fishery resources, but is nonetheless facing severe environmental challenges that have led to the decline of coastal habitats and fish diversity [31]. In order to formulate suitable conservation and restoration policies, it is particularly important to select appropriate investigation methods according to local environmental characteristics. Although eDNA is a very effective survey method for detecting fish community assemblages, which is not only economical but can also detect small, cryptic and nocturnal taxa [15], its use as an independent sampling method remains controversial [69,70]. As such, using a molecular eDNA method (e.g., metabarcoding) concurrently with a traditional fishing method (e.g., gillnet) appears to be the best way to investigate and monitor fish community abundance and diversity along mangrove–seagrass–coral reef continuums. In this study, the gillnet method enabled the physical collection of fish samples, provided more accurate habitat-specific fish community data and established new *12S* reference sequences, which collectively establish the foundation for improved future eDNA sampling. Due to the lower cost of eDNA sampling [71], it is more suitable for routine monitoring surveys for conservation management, because the generated molecular data supplements the physical gillnet community data to provide a more comprehensive understanding of the heterogeneity of fish taxonomic and functional diversity along mangrove–seagrass–coral reef continuums.

## 5. Conclusions

Our results confirm the ability of eDNA (i.e., contemporary molecular) and gillnet (i.e., traditional fishing) survey methods to detect fish communities in different habitats and to elucidate the heterogeneity of taxonomic and functional diversity along a mangrove–seagrass–coral reef continuum in Hainan, China. The fish communities detected within the sampled mangrove–seagrass–coral reef continuum were significantly different, although there were several genera that were common across all three habitats; however, considering the proximity of the sampled habitats within the continuum and the ability of fish to swim within and between the sampled habitats, we believe that connectivity exists to some extent between the continuum habitats. In order to improve the effectiveness of ecological management and protection, the findings of this study demonstrate that more comprehensive data can be obtained by combining eDNA metabarcoding and traditional gillnet survey methods than can be obtained by either survey method alone. The findings of this study also suggest that future conservation policies should consider the habitats within the mangrove–seagrass–coral reef continuum in Hainan, China as interconnected ecosystems rather than isolated habitats.

## Figures and Tables

**Table 1:** PERMANOVA results of fish assemblages at Wenchang, Hainan from South China based on method (eDNA vs. gillnet) and habitat (mangrove vs. seagrass vs. coral reef) as factors.

Source	Df	Pseudo-F	*p*
Habitat (eDNA)	2	0.25972	0.001
Habitat (gillnet)	2	4.8562	0.001
Method	1	0.06979	0.001

**Table 2:** The results of the SIMPER analysis based on detected fish genera using eDNA and gillnet methods across the sampled mangrove–seagrass–coral reef continuum.

Method	Habitats	Genera that Contributed the Greatest Dissimilarity		
eDNA & gillnet		*Plotosus* 70.3%	*Triacanthus* 67.8%	*Leiognathus* 65.0%
eDNA	Mangrove & Seagrass	Capoeta 71.7%	Pelates 68.5%	*Mugil* 64.7%
	Seagrass & Coral Reef	*Plotosus* 72.0%	*Capoeta* 69.5%	*Triacanthus* 66.6%
	Mangrove & Coral Reef	*Capoeta* 72.2%	*Plotosus* 69.5%	*Terapon* 66.6%
gillnet	Mangrove & Seagrass	*Terapon* 74.4%	*Gerres* 67.6%	*Eubleekeria* 58.8%
	Seagrass & Coral Reef	*Terapon* 70.1%	*Eubleekeria* 64.2%	*Lutjanus* 58.9%
	Mangrove & Coral Reef	*Plotosus* 73.6%	*Terapon* 69.7%	*Pelates* 65.1%

## Data Availability

The data presented in this study can be found in the Supplementary Materials.

## Supplementary Materials

The following supporting information can be downloaded at: https://www.mdpi.com/article/10.3390/ani13111777/s1, Table S1: Description of functional traits and categories. Table S2: Functional trait matrix for the six functional traits.
