# Supplementary material for: Heterogeneity of Fish Taxonomic and Functional Diversity Evaluated by eDNA and Gillnet along a Mangrove–Seagrass–Coral Reef Continuum
Source: Animals (Basel). 2023 May 26;13(11):1777. doi: 10.3390/ani13111777 (PMC10251956; doi:10.3390/ani13111777)
Supplement: Supplementary file 1 [file animals-13-01777-s001.zip › animals-2283502-supplementary.pdf]

Supplementary Materials of

Heterogeneity of Fish Taxonomic and Functional Diversity Evaluated by eDNA and Gillnet along a Mangrove–Seagrass–Coral Reef Continuum

Shuting Qiu [1,2,3], Jillian Lean Sim Ooi [4], Weilin Chen [2], Sze-Wan Poong [1], Han Zhang [2], Weiyi He [1,2], Shangke Su [2], Hao Luo [2], Wenjia Hu [2], Yang Amri Affendi [1], Jianguo Du [2,5,*] and Kar-Hoe Loh [1,*]

[1] Institute of Ocean and Earth Sciences, University of Malaya, Kuala Lumpur 50603, Malaysia
[2] Key Laboratory of Marine Ecological Conservation and Restoration, Third Institute of Oceanography, Ministry of Natural Resources, Xiamen 361005, China
[3] Institute for Advanced Study, University of Malaya, Kuala Lumpur 50603, Malaysia
[4] Department of Geography, Faculty of Arts and Social Sciences, University of Malaya, Kuala Lumpur 50603, Malaysia
[5] Faculty of Marine Biology, Xiamen Ocean Vocational College, Xiamen 36110, China
*Correspondence: dujianguo@tio.org.cn (J.D.) and khloh@um.edu.my (K.-H.L.)

Supplementary Table S1. Description of functional traits and categories.

**Trait**	**Description**	**Categories**
Trophic level	The position in the food chain is determined by the number of steps that energy is transferred to that level, from 2(for herbivores) to	2 (herbivore) to 4.5 (piscivore)
Maximum total length (cm)	The maximum length between the first and last points of the whole body, in a straight line, does not exceed the curve of the body.	Non-migratory<br>Restricted migratory<br>Long-distance migration

Mobility	The ability or tendency to move from one position or situation to another one	Sedentary<br>Mobile within reefs<br>Mobile across reefs
School size	The size of the fish population, generally measured by the number of individuals.	Solitary<br>Pairing<br>Small schools<br>Large schools
Water column position	Preferred vertical position in the water column for living and feeding.	Benthic (0-3 m)<br>Benthopelagic (3-10m)<br>Pelagic (10-30 m)<br>Demersal (>30 m)
Habitat type	Preferred habitats, do not mean that it does not occur in other habitats.	Estuarine<br>Stream<br>Mangrove<br>Seagrass<br>Coral Reed<br>Open area

Supplementary Table S2. Functional trait matrix for the six functional traits

Species	Trophic level	Maximum total length (cm)	Water column position	Mobility	Habitat type	school size
Abudefduf bengalensis	3.1	17.0	Benthic	Non-migratory	Coral reef	Large groups
Abudefduf sordidus	2.9	24.0	Benthic	Non-migratory	Coral reef	Solitary
Abudefduf vaigiensis	2.6	20.0	Benthopelagic	Long-distance migration	Coral reef	Large groups
Acanthopagrus australis	3.1	66.0	Demersal	Long-distance migration	Estuarine	Small groups
Acanthopagrus latus	3.8	40.0	Demersal	Long-distance migration	Estuarine	Small groups
Acanthopagrus pacificus	3.6	50.0	Pelagic	Restricted migratory	Estuarine	Small groups
Acanthopagrus schlegelii	3.2	50.0	Demersal	Restricted migratory	Estuarine	Small groups
Acanthopagrus sp.	3.8	40.0	Demersal	Restricted migratory	Estuarine	Small groups
Acentrogobius caninus	3.5	13.0	Benthic	Restricted migratory	Estuarine	Small groups
Acentrogobius multifasciatus	3.5	13.0	Demersal	Restricted migratory	Estuarine	Small groups
Acentrogobius pflaumii	3.1	12.0	Demersal	Non-migratory	Estuarine	Solitary
Alectis indica	4.1	165.0	Demersal	Long-distance migration	Coral reef	Large groups
Alepes djedaba	3.3	40.0	Demersal	Long-distance migration	Coral reef	Large groups
Alepes kleinii	3.5	18.2	Pelagic	Restricted migratory	Coral reef	Large groups
Alepes vari	3.7	56.0	Pelagic	Restricted migratory	Estuarine	Large groups
Aluterus scriptus	2.8	110.0	Demersal	Restricted migratory	Coral reef	Solitary
Ambassis buruensis	3.4	8.2	Benthic	Restricted migratory	Estuarine	Large groups
Ambassis gymnocephalus	3.9	8.2	Benthic	Restricted migratory	Estuarine	Large groups
Ambassis interrupta	2.7	12.0	Benthic	Long-distance migration	Estuarine	Large groups
Ambassis urotaenia	3.4	14.0	Demersal	Long-distance migration	Estuarine	Large groups

Species	Trophic level	Maximum total length (cm)	Water column position	Mobility	Habitat type	school size
Ambassis vachellii	3.3	6.5	Benthic	Long-distance migration	Estuarine	Large groups
Amblygobius phalaena	3.6	15.0	Pelagic	Restricted migratory	Coral reef	Pair
Amoya chusanensis	3.4	7.2	Demersal	Non-migratory	Estuarine	Small groups
Arothron hispidus	3.2	50.0	Pelagic	Non-migratory	Coral reef	Solitary
Asterropteryx semipunctata	2.4	6.5	Pelagic	Restricted migratory	Coral reef	Small groups
Atherinomorus pinguis	3.3	13.6	Benthopelagic	Restricted migratory	Coral reef	Small groups
Atherion elymus	3.4	6.4	Benthic	Non-migratory	Coral reef	Large groups
Aurigequula fasciata	3.3	21.0	Demersal	Restricted migratory	Estuarine	Large groups
Boleophthalmus sp.	2.0	17.5	Demersal	Non-migratory	Estuarine	Small groups
Bothus pantherinus	3.5	39.0	Demersal	Non-migratory	Coral reef	Small groups
Brachirus orientalis	3.5	38.0	Demersal	Long-distance migration	Estuarine	Small groups
Caffrogobius gilchristi	3.6	15.0	Demersal	Non-migratory	Estuarine	Small groups
Callionymus enneactis	3.3	8.0	Pelagic	Restricted migratory	Coral reef	Small groups
Callionymus meridionalis	3.3	10.8	Demersal	Non-migratory	Estuarine	Small groups
Callogobius tanegasimae	3.2	5.5	Demersal	Non-migratory	Estuarine	Small groups
Calotomus spinidens	2.0	30.0	Demersal	Restricted migratory	Coral reef	Small groups
Capoeta capoeta	2.0	30.0	Demersal	Restricted migratory	Coral reef	Small groups
Capoeta damascina	2.4	50.0	Pelagic	Restricted migratory	Coral reef	Small groups
Carangoides malabaricus	3.9	60.0	Demersal	Long-distance migration	Coral reef	Solitary
Carangoides praeustus	3.9	25.0	Demersal	Long-distance migration	Estuarine	Solitary
Caranx ignobilis	4.2	170.0	Demersal	Restricted migratory	Coral reef	Solitary
Caranx tille	4.1	80.0	Demersal	Non-migratory	Coral reef	Small groups

Species	Trophic level	Maximum total length (cm)	Water column position	Mobility	Habitat type	school size
Cephalopholis boenak	4.1	30.0	Demersal	Non-migratory	Coral reef	Pair
Chaetodon auriga	3.7	23.0	Demersal	Non-migratory	Coral reef	Small groups
Chaetodon lunula	3.7	20.0	Demersal	Restricted migratory	Coral reef	Pair
Chaetodon rafflesii	4.3	18.0	Pelagic	Restricted migratory	Coral reef	Pair
Chaetodon ulietensis	2.7	15.0	Demersal	Restricted migratory	Coral reef	Small groups
Chanos chanos	2.4	180.0	Pelagic	Long-distance migration	Estuarine	Small groups
Cheilinus chlorourus	3.5	45.0	Demersal	Restricted migratory	Coral reef	Small groups
Cheilio inermis	3.5	50.0	Demersal	Restricted migratory	Coral reef	Small groups
Chelonodontops patoca	3.1	38.0	Demersal	Long-distance migration	Coral reef	Small groups
Chirocentrus dorab	4.4	100.0	Demersal	Long-distance migration	Coral reef	Solitary
Chlorurus sordidus	2.6	40.0	Demersal	Long-distance migration	Coral reef	Solitary
Choerodon schoenleinii	3.4	100.0	Demersal	Restricted migratory	Coral reef	Solitary
Choerodon schoenleinii	3.4	100.0	Demersal	Long-distance migration	Coral reef	Solitary
Chrysiptera biocellata	2.0	12.5	Benthic	Non-migratory	Coral reef	Pair
Cirripectes polyzona	2.8	12.0	Demersal	Non-migratory	Coral reef	Pair
Cirripectes sp.	2.0	9.2	Benthic	Restricted migratory	Coral reef	Small groups
Clupanodon thrissa	3.1	26.0	Demersal	Long-distance migration	Estuarine	Large groups
Coryphaena hippurus	4.4	210.0	Pelagic	Long-distance migration	open area	Large groups
Crenimugil crenilabis	2.3	60.0	Pelagic	Non-migratory	Coral reef	Large groups
Crenimugil seheli	2.3	60.0	Benthic	Long-distance migration	Coral reef	Large groups
Crossorhombus azureus	3.5	18.0	Demersal	Non-migratory	Estuarine	Small groups
Cryptocentrus caeruleomaculatus	3.0	10.0	Benthic	Restricted migratory	Coral reef	Small groups

Species	Trophic level	Maximum total length (cm)	Water column position	Mobility	Habitat type	school size
Cryptocentrus nigrocellatus	3.4	13.0	Benthopelagic	Restricted migratory	Coral reef	Small groups
Dactyloptena gilberti	3.5	25.0	Demersal	Non-migratory	Estuarine	Small groups
Dactyloptena orientalis	3.7	40.0	Demersal	Non-migratory	Coral reef	Solitary
Dascyllus reticulatus	3.1	9.0	Demersal	Non-migratory	Coral reef	Large groups
Decapterus maruadsi	3.4	25.0	Demersal	Restricted migratory	Estuarine	Large groups
Dendrochirus zebra	4.0	25.0	Demersal	Long-distance migration	Coral reef	Small groups
Diodon liturosus	3.5	65.0	Demersal	Non-migratory	Coral reef	Solitary
Drombus globiceps	3.2	4.3	Demersal	Long-distance migration	Estuarine	Small groups
Drombus triangularis	3.3	7.0	Demersal	Long-distance migration	Estuarine	Small groups
Elops hawaiensis	4.0	120.0	Demersal	Long-distance migration	Estuarine	Large groups
Engraulis japonicus	3.1	18.0	Demersal	Long-distance migration	open area	Large groups
Enneapterygius unimaculatus	3.1	3.1	Benthic	Restricted migratory	Coral reef	Small groups
Entomacrodus caudofasciatus	2.0	6.2	Benthic	Non-migratory	Coral reef	Small groups
Entomacrodus decussatus	2.0	19.0	Benthic	Non-migratory	Coral reef	Small groups
Entomacrodus striatus	2.0	11.0	Benthic	Non-migratory	Coral reef	Small groups
Entomacrodus thalassinus	2.0	4.0	Benthic	Non-migratory	Coral reef	Small groups
Epinephelus bleekeri	3.9	76.0	Demersal	Restricted migratory	Estuarine	Solitary
Epinephelus coioides	4.0	120.0	Demersal	Restricted migratory	Coral reef	Solitary
Epinephelus fuscoguttatus	4.1	120.0	Demersal	Restricted migratory	Coral reef	Solitary
Epinephelus quoyanus	4.0	40.0	Demersal	Restricted migratory	Coral reef	Solitary
Equulites rivulatus	3.2	12.0	Demersal	Restricted migratory	Estuarine	Large groups
Escualosa thoracata	3.2	10.0	Demersal	Long-distance migration	Estuarine	Small groups

Species	Trophic level	Maximum total length (cm)	Water column position	Mobility	Habitat type	school size
Etroplus maculatus	2.7	9.5	Benthopelagic	Restricted migratory	Estuarine	Pair
Eubleekeria splendens	2.9	17.0	Demersal	Long-distance migration	Estuarine	Large groups
Eugnathogobius variegatus	3.4	4.0	Demersal	Non-migratory	Estuarine	Small groups
Euthynnus affinis	4.5	100.0	Demersal	Long-distance migration	open area	Large groups
Exallias brevis	4.3	14.5	Demersal	Non-migratory	Coral reef	Solitary
Favonigobius melanobranchus	3.5	8.3	Demersal	Restricted migratory	Seagrass	Small groups
Favonigobius reichei	3.5	8.3	Demersal	Long-distance migration	Estuarine	Small groups
Favonigobius sp.	3.5	8.3	Demersal	Long-distance migration	Estuarine	Small groups
Fistularia commersonii	4.3	160.0	Demersal	Restricted migratory	Coral reef	Small groups
Gazza minuta	4.2	21.0	Demersal	Restricted migratory	Estuarine	Small groups
Gazza rhombea	3.0	17.6	Demersal	Restricted migratory	Estuarine	Large groups
Gerres decacanthus	3.2	9.1	Benthopelagic	Restricted migratory	Estuarine	Large groups
Gerres erythrourus	3.3	30.0	Demersal	Long-distance migration	Coral reef	Large groups
Gerres filamentosus	3.3	39.0	Demersal	Long-distance migration	Estuarine	Large groups
Gerres japonicus	3.4	25.0	Demersal	Non-migratory	Estuarine	Small groups
Gerres longirostris	3.5	44.5	Demersal	Long-distance migration	Coral reef	Large groups
Gerres macracanthus	3.4	30.0	Demersal	Non-migratory	Estuarine	Large groups
Gerres oblongus	3.5	30.0	Demersal	Restricted migratory	Coral reef	Large groups
Gerres oyena	2.7	30.0	Pelagic	Restricted migratory	Coral reef	Small groups
Gerres shima	3.3	11.8	Benthopelagic	Restricted migratory	Estuarine	Large groups
Glossogobius giuris	3.7	50.0	Benthopelagic	Long-distance migration	Estuarine	Small groups
Gnatholepis cauerensis	2.3	6.0	Demersal	Non-migratory	Coral reef	Small groups

Species	Trophic level	Maximum total length (cm)	Water column position	Mobility	Habitat type	school size
Gobiopsis arenaria	3.0	1.6	Pelagic	Non-migratory	Coral reef	Small groups
Gymnothorax fimbriatus	4.0	93.4	Demersal	Restricted migratory	Coral reef	Solitary
Gymnothorax thyrsoideus	4.0	73.2	Demersal	Restricted migratory	Coral reef	Small groups
Gymnothorax undulatus	3.6	150.0	Demersal	Restricted migratory	Coral reef	Solitary
Halichoeres nigrescens	3.4	14.0	Benthopelagic	Long-distance migration	Coral reef	Small groups
Halichoeres nigrescens	3.4	14.0	Benthic	Long-distance migration	Coral reef	Small groups
Hapalogenys nigripinnis	4.0	40.0	Benthopelagic	Restricted migratory	Estuarine	Large groups
Helcogramma striata	3.4	4.3	Demersal	Non-migratory	Coral reef	Small groups
Helotes sexlineatus	2.0	15.0	Demersal	Restricted migratory	Estuarine	Small groups
Hemiculter leucisculus	2.8	23.0	Benthopelagic	Restricted migratory	Estuarine	Large groups
Hemigobius hoevenii	3.3	6.0	Benthic	Long-distance migration	Mangrove	Small groups
Hemiramphus lutkei	3.4	40.0	Demersal	Restricted migratory	Coral reef	Large groups
Hemitrygon bennettii	4.5	50.0	Demersal	Restricted migratory	Estuarine	Solitary
Hephaestus fuliginosus	2.8	54.0	Benthopelagic	Non-migratory	Stream	Small groups
Herklotsichthys quadrimaculatus	3.6	25.0	Benthic	Restricted migratory	Coral reef	Large groups
Hilsa kelee	2.9	30.0	Benthic	Long-distance migration	open area	Large groups
Hypoatherina temminckii	3.4	12.0	Benthic	Restricted migratory	Coral reef	Large groups
Hypoatherina tsurugae	3.4	15.0	Benthic	Restricted migratory	Estuarine	Large groups
Hypoatherina valenciennei	3.2	12.0	Benthic	Restricted migratory	Estuarine	Large groups
Hypoatherina woodwardi	3.4	10.0	Benthic	Restricted migratory	Coral reef	Large groups
Hyporhamphus dussumieri	3.5	38.0	Demersal	Restricted migratory	Coral reef	Large groups
Hyporhamphus limbatus	3.1	25.0	Benthic	Long-distance migration	Estuarine	Small groups

Species	Trophic level	Maximum total length (cm)	Water column position	Mobility	Habitat type	school size
Hyporhamphus quoyi	3.0	31.2	Benthopelagic	Restricted migratory	Estuarine	Large groups
Ilisha elongata	3.8	60.0	Pelagic	Restricted migratory	Estuarine	Small groups
Iniistius evides	3.4	19.0	Benthic	Non-migratory	Coral reef	Small groups
Istigobius campbelli	3.3	7.8	Pelagic	Non-migratory	Coral reef	Solitary
Istigobius goldmanni	3.3	6.0	Benthic	Non-migratory	Coral reef	Small groups
Istigobius ornatus	3.5	11.0	Benthic	Non-migratory	Mangrove	Solitary
Johnius dussumieri	4.1	40.0	Demersal	Long-distance migration	Estuarine	Small groups
Konosirus punctatus	2.9	32.0	Demersal	Long-distance migration	Estuarine	Large groups
Kyphosus elegans	2.9	53.0	Demersal	Non-migratory	Coral reef	Small groups
Kyphosus vaigiensis	2.0	70.0	Demersal	Long-distance migration	Coral reef	Large groups
Larimichthys crocea	3.7	80.0	Demersal	Restricted migratory	Estuarine	Small groups
Lates calcarifer	3.8	200.0	Demersal	Long-distance migration	Estuarine	Small groups
Lates japonicus	4.3	130.0	Demersal	Restricted migratory	Estuarine	Small groups
Leiognathus berbis	3.3	11.0	Demersal	Restricted migratory	Estuarine	Large groups
Leiognathus brevirostris	3.0	14.0	Demersal	Long-distance migration	Estuarine	Large groups
Leiognathus equula	3.0	28.0	Benthopelagic	Long-distance migration	Estuarine	Small groups
Leiognathus equulus	3.0	28.0	Demersal	Long-distance migration	Estuarine	Large groups
Leptoscarus vaigiensis	2.0	35.2	Pelagic	Restricted migratory	Coral reef	Small groups
Lethrinus haematopterus	3.7	45.0	Demersal	Non-migratory	Coral reef	Large groups
Lethrinus nebulosus	3.8	38.8	Pelagic	Non-migratory	Coral reef	Small groups
Lethrinus ornatus	3.4	45.0	Demersal	Non-migratory	Coral reef	Small groups
Lethrinus sp.	3.8	38.8	Pelagic	Non-migratory	Coral reef	Small groups

Species	Trophic level	Maximum total length (cm)	Water column position	Mobility	Habitat type	school size
Lethrinus xanthochilus	3.8	70.0	Demersal	Non-migratory	Coral reef	Solitary
Lethrinus haematopterus	3.7	45.0	Demersal	Non-migratory	Coral reef	Small groups
Liza carinata	2.6	18.0	Pelagic	Non-migratory	Estuarine	Large groups
Liza subviridis	2.7	40.0	Pelagic	Long-distance migration	Estuarine	Large groups
Lutjanus argentimaculatus	3.6	150.0	Demersal	Restricted migratory	Estuarine	Large groups
Lutjanus fulviflamma	3.8	35.0	Demersal	Restricted migratory	Coral reef	Large groups
Lutjanus lutjanus	4.1	35.0	Demersal	Restricted migratory	Coral reef	Large groups
Lutjanus russellii	4.1	80.0	Demersal	Restricted migratory	Coral reef	Large groups
Lutjanus argentimaculatus	3.6	150.0	Demersal	Long-distance migration	Coral reef	Large groups
Mastacembelus armatus	2.8	90.0	Demersal	Long-distance migration	Stream	Solitary
Monacanthus chinensis	2.4	38.0	Demersal	Restricted migratory	Coral reef	Pair
Moolgarda perusii	2.3	16.0	Demersal	Restricted migratory	Coral reef	Large groups
Moolgarda seheli	2.3	30.0	Demersal	Long-distance migration	Coral reef	Large groups
Mugil cephalus	2.5	100.0	Demersal	Long-distance migration	Estuarine	Large groups
Nematalosa come	2.8	21.0	Pelagic	Restricted migratory	Estuarine	Small groups
Nematalosa japonica	2.4	19.0	Benthopelagic	Restricted migratory	Estuarine	Large groups
Nematalosa nasus	2.2	25.5	Benthopelagic	Long-distance migration	Estuarine	Small groups
Neopomacentrus azysron	3.4	7.5	Pelagic	Non-migratory	Coral reef	Small groups
Neopomacentrus cyanomos	3.4	10.0	Demersal	Non-migratory	Coral reef	Large groups
Norfolkia thomasi	3.2	4.0	Pelagic	Restricted migratory	Coral reef	Small groups
Nuchequula longicornis	2.9	7.9	Demersal	Restricted migratory	Estuarine	Large groups
Nuchequula mannusella	2.9	14.0	Demersal	Restricted migratory	Estuarine	Large groups

Species	Trophic level	Maximum total length (cm)	Water column position	Mobility	Habitat type	school size
Oedalechilus labiosus	2.4	3.0	Benthic	Restricted migratory	Coral reef	Large groups
Omobranchus elongatus	2.7	5.0	Benthic	Non-migratory	Estuarine	Small groups
Oreochromis niloticus	2.0	60.0	Benthic	Long-distance migration	Estuarine	Solitary
Osteomugil formosae	2.4	25.0	Benthic	Long-distance migration	Coral reef	Large groups
Osteomugil perusii	2.4	25.0	Benthic	Long-distance migration	Coral reef	Large groups
Ostorhinchus cookii	3.5	10.0	Pelagic	Non-migratory	Coral reef	Small groups
Ostracion cubicum	3.4	45.0	Demersal	Restricted migratory	Coral reef	Solitary
Oxyurichthys sp.	4.2	17.0	Demersal	Long-distance migration	Estuarine	Small groups
Pagrus major	4.5	100.0	Demersal	Long-distance migration	open area	Large groups
Pampus argenteus	3.3	60.0	Demersal	Long-distance migration	Estuarine	Large groups
Parablennius yatabei	2.0	6.0	Pelagic	Long-distance migration	Estuarine	Small groups
Paracentropogon longispinis	3.2	13.0	Demersal	Non-migratory	Estuarine	Solitary
Paralichthys olivaceus	4.5	103.0	Demersal	Long-distance migration	Estuarine	Small groups
Parapercis cylindrica	3.0	23.0	Pelagic	Non-migratory	Coral reef	Solitary
Parapristipoma trilineatum	3.4	40.0	Demersal	Restricted migratory	Coral reef	Large groups
Parascorpaena mossambica	3.6	20.0	Pelagic	Non-migratory	Coral reef	Solitary
Parupeneus ciliatus	3.5	38.0	Demersal	Restricted migratory	Coral reef	Small groups
Parupeneus cyclostomus	4.2	50.0	Demersal	Non-migratory	Coral reef	Solitary
Parupeneus indicus	3.5	45.0	Pelagic	Non-migratory	Coral reef	Small groups
Pelates quadrilineatus	3.6	30.0	Pelagic	Restricted migratory	Estuarine	Large groups
Pelates sexlineatus	2.0	32.0	Pelagic	Restricted migratory	Estuarine	Large groups
Pelates quadrilineatus	3.6	30.0	Demersal	Restricted migratory	Coral reef	Large groups

Species	Trophic level	Maximum total length (cm)	Water column position	Mobility	Habitat type	school size
Pempheris adusta	3.4	17.0	Pelagic	Non-migratory	Coral reef	Small groups
Pempheris schwenkii	3.4	40.0	Demersal	Non-migratory	Coral reef	Small groups
Pennahia aneus	4.0	30.0	Demersal	Restricted migratory	Estuarine	Small groups
Petroscirtes mitratus	2.0	8.5	Benthic	Non-migratory	Coral reef	Small groups
Petroscirtes variabilis	3.1	15.0	Benthic	Non-migratory	Coral reef	Small groups
Photopectoralis bindus	2.9	11.0	Demersal	Long-distance migration	Estuarine	Large groups
Pisodonophis cancrivorus	3.8	108.0	Demersal	Non-migratory	Coral reef	Solitary
Planiliza carinata	2.5	24.4	Pelagic	Restricted migratory	Estuarine	Large groups
Planiliza subviridis	2.7	40.0	Benthopelagic	Long-distance migration	Estuarine	Large groups
Platycephalus indicus	3.6	100.0	Demersal	Long-distance migration	Coral reef	Small groups
Plectorhinchus lineatus	3.9	72.0	Demersal	Restricted migratory	Coral reef	Pair
Plectroglyphidodon leucozonus	2.0	12.0	Benthic	Non-migratory	Coral reef	Small groups
Plectroglyphidodon obreptus	2.0	12.0	Benthic	Non-migratory	Coral reef	Pair
Plectropomus leopardus	4.4	120.0	Demersal	Non-migratory	Coral reef	Solitary
Plesiops nakaharae	3.5	14.0	Demersal	Non-migratory	Coral reef	Small groups
Plicomugil labiosus		40.0	Benthic	Restricted migratory	Coral reef	Large groups
Plotosus lineatus	3.6	20.0	Demersal	Long-distance migration	Coral reef	Large groups
Plotosus lineatus	3.9	50.0	Demersal	Long-distance migration	Estuarine	Large groups
Polydactylus plebeius	3.6	45.0	Demersal	Restricted migratory	Estuarine	Large groups
Pomacentrus chrysurus	2.6	9.0	Benthic	Non-migratory	Coral reef	Small groups
Pomacentrus tripunctatus	2.0	7.5	Benthic	Non-migratory	Coral reef	Solitary
Pomadasys maculatus	4.0	59.3	Demersal	Long-distance migration	Coral reef	Pair

Species	Trophic level	Maximum total length (cm)	Water column position	Mobility	Habitat type	school size
Psammogobius biocellatus	3.4	12.0	Benthopelagic	Long-distance migration	Estuarine	Small groups
Psenopsis anomala	4.0	30.0	Demersal	Restricted migratory		Small groups
Pseudetroplus maculatus	2.4	40.0	Demersal	Restricted migratory	Coral reef	Small groups
Pseudochromis cyanotaenia	3.3	6.0	Demersal	Non-migratory	Coral reef	Pair
Pseudogobius javanicus	4.2	6.0	Benthopelagic	Restricted migratory	Estuarine	Small groups
Pseudogobius masago	3.1	2.5	Demersal	Non-migratory	Estuarine	Small groups
Pseudogobius poicilosoma	3.3	6.5	Demersal	Long-distance migration	Estuarine	Small groups
Pseudorhombus dupliciocellatus	4.5	40.0	Demersal	Non-migratory	Estuarine	Small groups
Pteragogus flagellifer	3.5	20.0	Demersal	Non-migratory	Coral reef	Small groups
Ptereleotris microlepis	3.4	13.0	Demersal	Non-migratory	Coral reef	Pair
Rachycentron canadum	4.0	200.0	Demersal	Long-distance migration	Coral reef	Solitary
Rastrelliger kanagurta	3.2	38.0	Demersal	Long-distance migration	open area	Large groups
Rhynchoconger ectenurus	4.0	65.0	Demersal	Non-migratory	Estuarine	Solitary
Rhynchopelates oxyrhynchus	3.6	25.0	Pelagic	Restricted migratory	Estuarine	Large groups
Rogadius asper	3.6	17.0	Demersal	Long-distance migration	Estuarine	Small groups
Salarias fasciatus	2.0	14.0	Benthic	Restricted migratory	Coral reef	Small groups
Sardinella albella	2.6	15.0	Demersal	Restricted migratory	Coral reef	Large groups
Sardinella hualiensis	2.9	12.5	Demersal	Restricted migratory	Estuarine	Large groups
Scarus ghobban	2.0	90.0	Demersal	Restricted migratory	Coral reef	Large groups
Scarus sp.	2.0	90.0	Demersal	Restricted migratory	Coral reef	Large groups
Scatophagus argus	3.0	38.0	Benthic	Long-distance migration	Coral reef	Large groups
Scolopsis ciliata	3.7	25.0	Pelagic	Restricted migratory	Coral reef	Small groups

Species	Trophic level	Maximum total length (cm)	Water column position	Mobility	Habitat type	school size
Scolopsis lineata	3.8	23.0	Pelagic	Restricted migratory	Coral reef	Small groups
Scolopsis monogramma	3.5	38.0	Demersal	Non-migratory	Coral reef	Small groups
Scolopsis vosmeri	3.5	25.0	Demersal	Non-migratory	Coral reef	Small groups
Scomberoides lysan	4.0	110.0	Demersal	Restricted migratory	Coral reef	Small groups
Scomberoides tol	4.1	60.0	Pelagic	Restricted migratory	Coral reef	Small groups
Scorpaenopsis diabolus	4.2	30.0	Demersal	Restricted migratory	Coral reef	Solitary
Scorpaenopsis macrochir	3.9	13.6	Demersal	Restricted migratory	Coral reef	Pair
Scorpaenopsis neglecta	4.1	19.0	Demersal	Non-migratory	Coral reef	Solitary
Scorpaenopsis possi	4.1	19.4	Demersal	Non-migratory	Coral reef	Solitary
Selenotoca multifasciata	2.9	40.0	Benthic	Long-distance migration	Estuarine	Small groups
Setipinna taty	3.6	22.0	Demersal	Restricted migratory	Estuarine	Large groups
Siganus canaliculatus	2.8	40.0	Demersal	Long-distance migration	Coral reef	Small groups
Siganus fuscescens	2.0	40.0	Demersal	Long-distance migration	Coral reef	Small groups
Siganus guttatus	2.7	42.0	Benthic	Restricted migratory	Coral reef	Large groups
Siganus sutor	2.3	45.0	Demersal	Restricted migratory	Coral reef	Large groups
Sillago aeolus	3.3	30.0	Demersal	Non-migratory	Coral reef	Large groups
Sillago maculata	3.3	30.0	Demersal	Non-migratory	Coral reef	Small groups
Sillago sihama	3.3	31.0	Demersal	Long-distance migration	Coral reef	Large groups
Sparus aurata	3.7	70.0	Demersal	Non-migratory	Estuarine	Small groups
Sphyraena jello	4.5	150.0	Demersal	Long-distance migration	Coral reef	Solitary
Spratelloides delicatulus	3.1	7.0	Demersal	Restricted migratory	Coral reef	Large groups
Stegastes obreptus	2.0	12.0	Benthic	Non-migratory	Coral reef	Small groups

Species	Trophic level	Maximum total length (cm)	Water column position	Mobility	Habitat type	school size
Stephanolepis cirrhifer	2.8	20.0	Demersal	Long-distance migration	Estuarine	Large groups
Stethojulis terina	3.5	12.6	Benthic	Non-migratory	Coral reef	Small groups
Stolephorus commersonnii	3.1	10.0	Pelagic	Long-distance migration	Estuarine	Large groups
Stolephorus indicus	3.6	15.5	Demersal	Long-distance migration	Estuarine	Large groups
Strongylura incisa	4.2	100.0	Benthic	Restricted migratory	Coral reef	Small groups
Strongylura strongylura	4.2	40.0	Pelagic	Restricted migratory	Estuarine	Small groups
Strophidon sathete	4.0	400.0	Demersal	Restricted migratory	Coral reef	Solitary
Synechogobius ommaturus	3.8	43.0	Demersal	Restricted migratory	Estuarine	Small groups
Taeniamia fucata	3.5	8.0	Demersal	Restricted migratory	Coral reef	Large groups
Taenioides cirratus	3.9	30.0	Demersal	Long-distance migration	Estuarine	Small groups
Takifugu alboplumbeus	3.3	23.0	Demersal	Restricted migratory	Estuarine	Solitary
Tatia intermedia	3.5	12.0	Benthopelagic	Non-migratory	Stream	Solitary
Terapon jarbua	3.9	36.0	Pelagic	Long-distance migration	Estuarine	Large groups
Terapon theraps	3.5	30.0	Demersal	Restricted migratory	Coral reef	Large groups
Thalassoma bifasciatum	3.3	25.0	Demersal	Restricted migratory	Coral reef	Small groups
Thalassoma lunare	3.5	25.0	Pelagic	Non-migratory	Coral reef	Small groups
Thryssa hamiltonii	3.5	27.0	Pelagic	Long-distance migration	Estuarine	Large groups
Thryssa hamiltonii	3.5	27.0	Pelagic	Long-distance migration	Estuarine	Small groups
Trachinocephalus myops	4.4	25.0	Demersal	Non-migratory	Coral reef	Small groups
Trachinotus carolinus	3.5	64.0	Demersal	Long-distance migration	Estuarine	Large groups
Triacanthus biaculeatus	2.8	30.0	Demersal	Restricted migratory	Estuarine	Small groups
Trichiurus lepturus	4.4	234.0	Demersal	Long-distance migration	open area	Large groups

Species	Trophic level	Maximum total length (cm)	Water column position	Mobility	Habitat type	school size
Tylosurus acus	4.5	153.0	Demersal	Long-distance migration	open area	Small groups
Tylosurus crocodilus	4.4	150.0	Pelagic	Long-distance migration	Coral reef	Large groups
Upeneus guttatus	3.5	16.0	Demersal	Non-migratory	Estuarine	Small groups
Upeneus tragula	3.6	25.0	Demersal	Long-distance migration	Coral reef	Solitary
Valamugil formosae	2.2	30.0	Demersal	Restricted migratory	Coral reef	Large groups
Yongeichthys criniger	3.4	15.0	Demersal	Non-migratory	Estuarine	Small groups
Zenarchopterus buffonis	2.5	23.0	Pelagic	Restricted migratory	Coral reef	Large groups
Zenarchopterus dunckeri	3.2	14.0	Benthic	Restricted migratory	Estuarine	Large groups
